# G1 checkpoint protein and p53 abnormalities occur in most invasive transitional cell carcinomas of the urinary bladder

**DOI:** 10.1038/sj.bjc.6990483

**Published:** 1999-06

**Authors:** G A Niehans, R A Kratzke, M K Froberg, D M Aeppli, P L Nguyen, J Geradts

**Affiliations:** Departments of Pathology, Medicine, and Biostatistics, Minneapolis Department of Veterans Affairs Medical Center, 1 Veterans Drive, Minneapolis, MN, 55417, USA; Department of Pathology and Laboratory Medicine, University of North Carolina School of Medicine, Chapel Hill, NC, 27599, USA

**Keywords:** bladder neoplasms, cyclin D1, retinoblastoma protein, p16^INK4^, p53

## Abstract

The G1 cell cycle checkpoint regulates entry into S phase for normal cells. Components of the G1 checkpoint, including retinoblastoma (Rb) protein, cyclin D1 and p16^INK4a^, are commonly altered in human malignancies, abrogating cell cycle control. Using immunohistochemistry, we examined 79 invasive transitional cell carcinomas of the urinary bladder treated by cystectomy, for loss of Rb or p16^INK4a^ protein and for cyclin D1 overexpression. As p53 is also involved in cell cycle control, its expression was studied as well. Rb protein loss occurred in 23/79 cases (29%); it was inversely correlated with loss of p16^INK4a^, which occurred in 15/79 cases (19%). One biphenotypic case, with Rb+p16– and Rb-p16+ areas, was identified as well. Cyclin D1 was overexpressed in 21/79 carcinomas (27%), all of which retained Rb protein. Fifty of 79 tumours (63%) showed aberrant accumulation of p53 protein; p53 staining did not correlate with Rb, p16^INK4a^, or cyclin D1 status. Overall, 70% of bladder carcinomas showed abnormalities in one or more of the intrinsic proteins of the G1 checkpoint (Rb, p16^INK4a^ and cyclin D1). Only 15% of all bladder carcinomas (12/79) showed a normal phenotype for all four proteins. In a multivariate survival analysis, cyclin D1 overexpression was linked to less aggressive disease and relatively favourable outcome. In our series, Rb, p16^INK4a^ and p53 status did not reach statistical significance as prognostic factors. In conclusion, G1 restriction point defects can be identified in the majority of bladder carcinomas. Our findings support the hypothesis that cyclin D1 and p16^INK4a^ can cooperate to dysregulate the cell cycle, but that loss of Rb protein abolishes the G1 checkpoint completely, removing any selective advantage for cells that alter additional cell cycle proteins. © 1999 Cancer Research Campaign


*British Journal of Cancer* (1999) **80**(8), 1175–1184
© 1999 Cancer Research Campaign
Article no. bjoc.1998.0483

### G1 checkpoint protein and p53 abnormalities occur in most invasive transitional cell carcinomas of the urinary bladder

**GA Niehans[1], RA Kratzke[2], MK Froberg[1]*, DM Aeppli[3], PL Nguyen[1] and J Geradts[4,†]**

Departments of [1]Pathology, [2]Medicine, and [3]Biostatistics, Minneapolis Department of Veterans Affairs Medical Center, 1 Veterans Drive, and University of Minnesota Medical School, Minneapolis, MN 55417, USA; [4]Department of Pathology and Laboratory Medicine, University of North Carolina School of Medicine, Chapel Hill, NC 27599, USA

**Summary** The G1 cell cycle checkpoint regulates entry into S phase for normal cells. Components of the G1 checkpoint, including retinoblastoma (Rb) protein, cyclin D1 and p16$^{INK4a}$, are commonly altered in human malignancies, abrogating cell cycle control. Using immunohistochemistry, we examined 79 invasive transitional cell carcinomas of the urinary bladder treated by cystectomy, for loss of Rb or p16$^{INK4a}$ protein and for cyclin D1 overexpression. As p53 is also involved in cell cycle control, its expression was studied as well. Rb protein loss occurred in 23/79 cases (29%); it was inversely correlated with loss of p16$^{INK4a}$, which occurred in 15/79 cases (19%). One biphenotypic case, with Rb+p16– and Rb-p16+ areas, was identified as well. Cyclin D1 was overexpressed in 21/79 carcinomas (27%), all of which retained Rb protein. Fifty of 79 tumours (63%) showed aberrant accumulation of p53 protein; p53 staining did not correlate with Rb, p16$^{INK4a}$, or cyclin D1 status. Overall, 70% of bladder carcinomas showed abnormalities in one or more of the intrinsic proteins of the G1 checkpoint (Rb, p16$^{INK4a}$ and cyclin D1). Only 15% of all bladder carcinomas (12/79) showed a normal phenotype for all four proteins. In a multivariate survival analysis, cyclin D1 overexpression was linked to less aggressive disease and relatively favourable outcome. In our series, Rb, p16$^{INK4a}$ and p53 status did not reach statistical significance as prognostic factors. In conclusion, G1 restriction point defects can be identified in the majority of bladder carcinomas. Our findings support the hypothesis that cyclin D1 and p16$^{INK4a}$ can cooperate to dysregulate the cell cycle, but that loss of Rb protein abolishes the G1 checkpoint completely, removing any selective advantage for cells that alter additional cell cycle proteins.

**Keywords:** bladder neoplasms; cyclin D1; retinoblastoma protein; p16$^{INK4}$; p53

To acquire a neoplastic phenotype, cells must disable multiple regulatory mechanisms including cell cycle control (Strauss et al, 1995). Abrogation of the G1 cell cycle checkpoint occurs in many malignancies. Key components of the checkpoint include retinoblastoma protein (Rb), cyclin D1 (also known as bcl-1 and PRAD-1), and the cyclin-dependent kinase inhibitor p16$^{INK4a}$. In normal cells, the Rb protein acts as a cell cycle brake while in its active, hypophosphorylated state. Cyclin D1 is a short-lived protein with a half-life of 30 min or less. When cyclin D1 is induced in early G1 by mitogenic signals such as growth factors, it binds to and activates the cyclin-dependent kinases CDK4 and CDK6, and these cyclin–kinase complexes then phosphorylate and inactivate the Rb protein, allowing the cell to proceed into S phase (reviewed by Strauss et al, 1995). p16$^{INK4a}$ binds to CDK–cyclin complexes and blocks phosphorylation of the Rb protein. Since inactivation of Rb enhances transcription of p16$^{INK4a}$, p16$^{INK4a}$ may act in normal cells as part of a negative feedback loop to turn off CDK4–cyclin D activity once the cell has passed the G1 restriction point (Serrano et al, 1993; Li et al, 1994; Parry et al, 1995). Thus, inactivation of either p16$^{INK4a}$ or Rb protein shifts the balance toward continued cell proliferation, as does amplification/overexpression of cyclin D1.

*Received 8 July 1998*
*Accepted 23 November 1998*

*Correspondence to:* GA Niehans

Abnormalities of individual components of the Rb/cyclin D1/p16$^{INK4a}$ pathway have been previously reported in cases of transitional cell carcinoma of the bladder (Cordon-Cardo et al, 1992; Logothetis et al, 1992; Geradts et al, 1995; Gruis et al, 1995; Orlow et al, 1995; Packenham et al, 1995; Williamson et al, 1995; Bringuier et al, 1996; Lee et al, 1997; Shin et al, 1997). In order to assess the overall frequency of G1 checkpoint defects in bladder carcinoma, and to examine the interrelationship between abnormalities in component proteins, we studied a series of 79 transitional cell carcinomas for evidence of cyclin D1 overexpression or loss of Rb or p16$^{INK4a}$ proteins, using immunohistochemistry. The p53 status of these tumours was also assessed via immunohistochemistry, since p53 influences cell cycle control through an independent pathway mediated by p21$^{WAF1/CIP1}$ (El-Deiry et al, 1993).

### MATERIALS AND METHODS

### Case selection and patient information

Most cases (77 of 79) were drawn from a previously published series (Nguyen et al, 1994) of 91 invasive transitional cell carci-

*Present Address: Department of Pathology and Laboratory Medicine, University of Minnesota School of Medicine, Duluth, MN, USA

†Present Address: Nuffield Department of Pathology and Bacteriology, University of Oxford, John Radcliffe Hospital, Oxford, UK

Presented in part at the 87th Annual Meeting of the US and Canadian Academy of Pathology, 28 February to 6 March 1998, in Boston, MA, USA

**Table 1** Clinical and pathologic characteristics of 79 invasive transitional cell carcinomas of the urinary bladder treated by cystectomy

| | | |
|---|---|---|
| Mean age (years) at time of diagnosis | 64.7, range 48–82 | (median 65) |
| Mean follow-up (months) since surgery (survivors) | 100.6, range 0–214 | (median 94) |
| Mean follow-up (months) since surgery (patients who died during study) | 44.1, range 1–134 | (median 57) |
| Pathologic stage[a] of tumours [TNM] (*n* = 79) | | |
| I[b] | 15 | |
| II | 18 | |
| III | 23 | |
| IV | 23 | |
| Pathologic grade of tumours [Ash] (*n* = 79) | | |
| 1 | 0 | |
| 2 | 2 | |
| 3 | 54 | |
| 4 | 23 | |
| EGFR index (*n* = 77)[c] | | |
| High | 28 | |
| Low | 49 | |
| PCNA percentage (*n* = 76)[a,c] | | |
| High | 42 | |
| Low | 34 | |
| DNA ploidy status (*n* = 61)[c] | | |
| Diploid | 30 | |
| Aneuploid | 31 | |
| S phase fraction (*n* = 35)[c] | | |
| High | 14 | |
| Low | 21 | |

EGFR, epidermal growth factor receptor; PCNA, proliferating cell nuclear antigen. [a]Tumours were staged according to the American Joint Committee on Cancer (1992) *Manual for Staging Cancer*, 4th edn. [b]All Stage 1 tumours in this study were T1 lesions. Non-invasive Ta/Tis lesions were excluded. [c]EGFR index, PCNA percentage, DNA ploidy status, and S-phase fraction were determined in a previous publication (Nguyen et al, 1994).

nomas of the urinary bladder treated by cystectomy at the Minneapolis Department of Veterans Affairs Medical Center between April 1975 and December 1989. Fourteen of the original 91 cases did not have sufficient residual tumour tissue in paraffin blocks to allow for further studies. Two new cases were added to the current series because of the acquisition of clinical and follow-up data not available at the time that the previous series was assembled. Nine cases were included in a previous report examining Rb and p16$^{INK4a}$ expression in archival human solid tumours (Geradts et al, 1995). All cases were staged according to the TNM system as specified by the American Joint Committee on Cancer (American Joint Committee on Cancer, 1992), and assigned a histological grade of 2–4 using Ash's criteria as outlined by Rosai (1996). Grade 1 was not assigned in this series since all tumours were invasive, and since only two carcinomas were assigned a grade of 2, grades 2 and 3 carcinomas were considered together in analyses. Seventeen patients had received adjuvant perioperative radiation therapy, 13 patients received adjuvant chemotherapy, and 17 received both. Because the administration of adjuvant therapy was nonrandomized (i.e. it was prescribed at the discretion of individual physicians for patients whom they judged to be at high risk of recurrence), and because a wide variety of regimens were employed, we did not stratify patient groups according to adjuvant status in the survival analysis. We did test adjuvant and non-adjuvant groups for imbalances with regards to cell cycle variables, to look for a source of introduced bias. Adjuvant and non-adjuvant groups were balanced with respect to p53, Rb, p16 and p16+Rb status. Adjuvant therapy was less likely to have been administered to patients with cyclin D1-overexpressing tumours, presumably because of the association between cyclin D1 overexpression and lower tumour grade (see Results and Discussion sections below). If adjuvant therapy were expected to improve survival, then the observed imbalance would strengthen and not weaken the association between cyclin D1 overexpression and favourable prognosis.

The mean patient age, mean and median follow-up times after surgery, and stage and grade of the 79 cases are shown in Table 1. Patient information was obtained from the Minneapolis Veterans Administration Medical Center Tumour Registry and from chart review. All cases were transitional cell carcinomas; other morphologies were excluded at the time of the selection of the original series. Seventy-eight patients were male and one was female (reflecting the patient population served at our institution). Only three living patients were lost to follow-up during the course of the study (at 0, 10 and 46 months respectively). There were four post-operative deaths due to infection or perioperative myocardial infarct, with no residual cancer at the time of death. Twenty-seven additional patients died of causes unrelated to bladder cancer during the course of the study, with four of those deaths occurring

 *© 1999 Cancer Research Campaign*

during the first year after cystectomy. Twenty-five patients died with or due to bladder cancer. Finally, 20 patients were alive and being followed at the time that the study concluded; no patients were living with disease. The follow-up period for these disease-free survivors ranged from 64 to 214 months (mean 112.9 months, median 95 months). The mean follow-up time for all survivors (including those lost to follow-up while alive) was 100.6 months (median, 94 months). The length of follow-up for patients who died during the course of the study ranged from 1 to 134 months (mean, 44.1 months, median 28.5 months).

Drawing on the results of our previous study (Nguyen et al, 1994), information on epidermal growth factor receptor (EGFR) status was available for 77 cases, percentage of proliferating cell nuclear antigen (PCNA)-positive tumour cells for 76 cases, ploidy status for 61 cases, and S phase fraction for 35 cases. These data are also presented in Table 1.

### Immunohistochemistry

The Rb antibody used in this study, 3C8, was obtained from Canji Inc. (San Diego, CA, USA), and used at a dilution of 1 μg $ml^{-1}$ for 1 h, following an epitope retrieval step using hot citrate buffer. Immunohistochemical staining was carried out as previously described (Geradts et al, 1996). A polyclonal $p16^{INK4a}$ antiserum obtained from PharMingen (San Diego, CA, USA), was applied to all bladder carcinomas, at a dilution of 1:400 overnight, using a previously described protocol (Geradts et al, 1995). For equivocal cases, follow-up $p16^{INK4a}$ staining was performed with a monoclonal antibody, G175-405 (2 μg $ml^{-1}$), from PharMingen; tissues were incubated overnight with the antibody after epitope retrieval with hot citrate buffer. p53 immunostaining utilized the D07 antibody (Dako, Carpinteria, CA, USA) at a dilution of 1:200, using a previously published procedure (Resnick et al, 1995). A cyclin D1 antibody (clone P2D11F11) was obtained from Vector Laboratories (Burlingame, CA, USA acting as US distributor for Novocastra Laboratories, Newcastle-upon-Tyne, UK) and used at a dilution of 1:1000. For cyclin D1, we utilized epitope retrieval in which deparaffinized and rehydrated 4-micron tissue sections were placed in Coplin jars containing 10 mM citrate buffer, pH 6, and boiled for 10 min in a 6-liter pressure cooker (Decor USA, Palatine, IL, USA) at 12 pounds per square inch. After cooling, the slides were rinsed in 0.01 M phosphate-buffered saline (PBS), pH 7.4, and incubated for 15 min with 15 μl $ml^{-1}$ horse serum to block non-specific binding. After an overnight incubation at 4°C with the primary antibody, the slides were immunolabelled by an avidin–biotin–peroxidase complex technique, utilizing the Vector mouse Elite ABC kit (Vector Laboratories, Burlingame, CA, USA), according to manufacturers' instructions. Chromogenic development was accomplished by incubation with 0.5 mg $ml^{-1}$ 3,3′-diaminobenzidine tetrahydrochloride (DAB) containing 0.009% hydrogen peroxide, and terminated after 5 min by a tap water rinse. Slides were then counterstained in haematoxylin and coverslipped.

The following external positive controls were used: mesothelioma cell line H2373 for Rb (p16–Rb+); lung cancer cell lines H417 and H2009 (p16+Rb–), as well as a nude mouse xenograft of H2009 cells, for $p16^{INK4a}$. In addition, non-neoplastic cells provided positive internal controls in every section of tumour. A lung carcinoma with a known mis-sense mutation in p53 was utilized as a p53-positive control, while a cyclin D1 overexpressing squamous carcinoma of the head and neck served as a control for cyclin D1. In addition, sections were reacted with non-specific mouse IgG and rabbit serum, respectively, as negative controls.

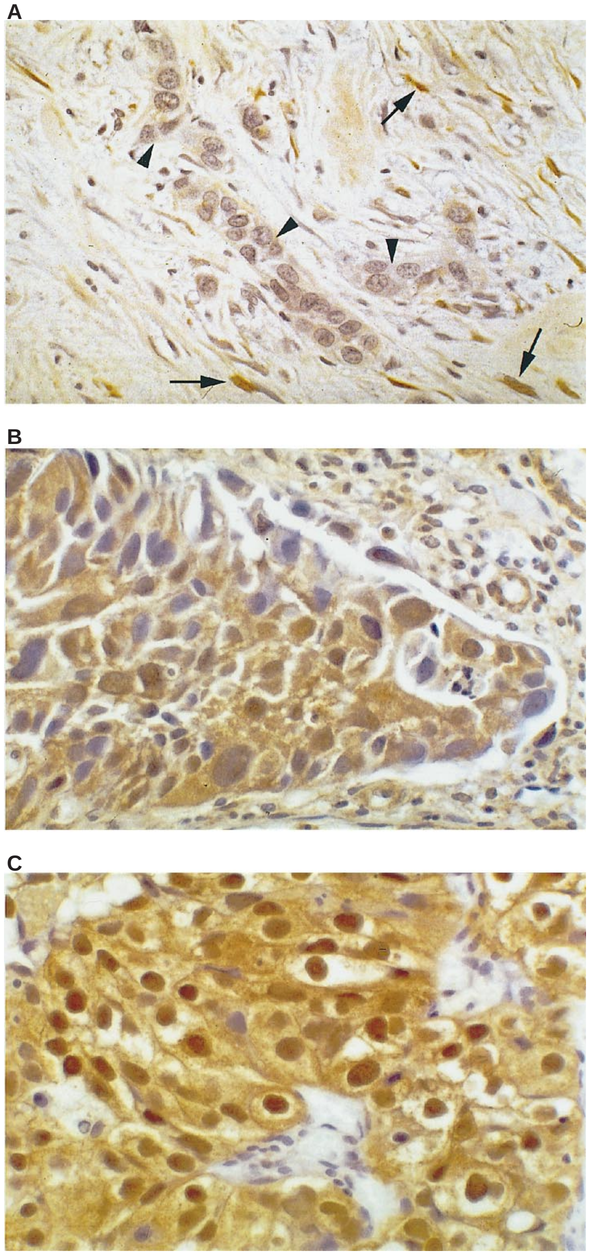

**Figure 1** Patterns of $p16^{INK4a}$ expression in transitional cell carcinomas. (**A**) p16 -negative tumour. Absence of staining in invasive carcinoma (arrow heads) with preserved reactivity in surrounding stromal cells (arrows). (**B**) p16-positive tumour ('low'). Note weak to moderate nuclear staining in less than 50% of neoplastic cells, associated with moderate cytoplasmic background reactivity. (**C**) p16-positive tumour ('high'). Note strong nuclear staining in over 90% of neoplastic cells, associated with moderate cytoplasmic background reactivity. Original magnification 400× (**A–C**)

### Interpretation of stains

All immunohistochemical stains were evaluated independently by two pathologists (GN and JG evaluated Rb, $p16^{INK4a}$ and p53 stains, while GN and KF evaluated cyclin D1 slides). Differences in interpretation were reconciled by rereview of slides separately or jointly at a double-headed microscope. Rb and $p16^{INK4a}$ were evaluated

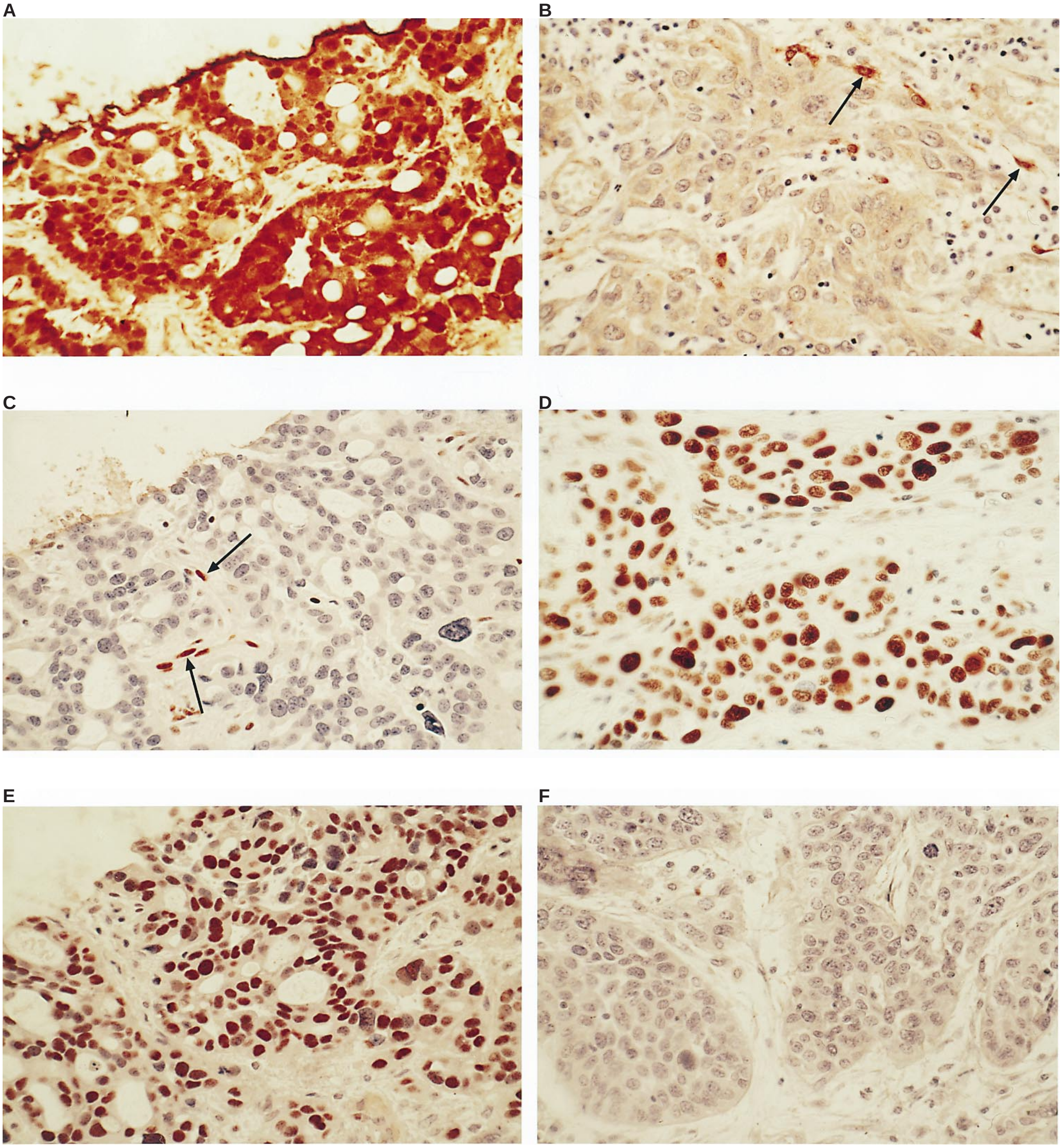

**Figure 2** Tumour suppressor gene expression in a biphenotypic muscle invasive transitional cell carcinoma. Left panels: one area near the surface of the tumour; right panels: a different area closer to the centre of the tumour. (**A, B**) p16$^{INK4a}$ stains. Note absence of nuclear staining in the tumour cells in (**B**), with strong nuclear and cytoplasmic staining (dark brown) in admixed stromal cells (arrows). (**C, D**) Rb stains. In (**C**), the tumour is completely negative, while normal endothelial cells show strong nuclear staining (arrows). (**E, F**) p53 stains. In (**E**), most tumour cells show strong nuclear overexpression. Original magnification 400× (**A–F**)

using previously described criteria (Geradts et al, 1995; Geradts et al, 1996). Only nuclear staining was evaluated; cytoplasmic reactivity, if present, was disregarded. A tumour was considered normal (positive) for Rb or p16$^{INK4a}$ if there was distinct nuclear staining in all areas of the lesion. A tumour was considered abnormal if there was no definite nuclear staining within the malignant cells *with* preserved nuclear reactivity in admixed non-neoplastic cells. In a small number of tumours, loss of Rb or p16$^{INK4a}$ was restricted to a defined area within the lesion, suggestive of intratumoural heterogeneity. These cases were classified as abnormal as well. If neither tumour nor normal cells showed definite nuclear reactivity above any cytoplasmic background, the case was considered equivocal. Cases were judged to show aberrant accumulation of p53 when 10% or more of tumour cell nuclei displayed moderate to strong immunoreactivity for p53 (Esrig et al, 1994). Slides stained for cyclin D1 were graded as low expression (< 5% of tumour cell nuclei showing weak immunoreactivity) or strong expression (5% or more of tumour cell nuclei showing moderate to strong

 © 1999 Cancer Research Campaign

**Table 2** Correlation of Rb and p16 or cyclin D1 expression

| | | p16[a] | | cyclin D1 | |
|---|---|---|---|---|---|
| | | N | A | N | A |
| Rb | N | 39 | 15 | 34 | 21 |
| | A | 23 | 0 | 24 | 0 |
| | | *P* = 0.004[b] | | *P* = 0.0002[b] | |

N = normal expression; A = abnormal expression (loss of expression for RB, p16; high level of expression for cyclin D1). [a]One additional case was indeterminate for p16$^{INK4a}$, and one case was biphenotypic (not included in this analysis). [b]*P*-values were determined by Fisher's exact test.

**Table 3** Cell cycle proteins and invasive bladder carcinomas: percentage of cases with abnormalities of cell cycle-associated and tumour suppressor proteins

| Variable | Normal | Abnormal | Equivocal |
|---|---|---|---|
| p53 | 29 (37%) | 50 (63%) | – |
| Rb | 55 (70%) | 24[d] (30%) | – |
| p16 | 62 (79%) | 16[d] (20%) | 1 (1%) |
| p16+Rb[a] | 39 (49.5%) | 39 (49.5%) | 1 (1%) |
| cyclin D1 | 58 (73%) | 21 (27%) | – |
| p16+Rb+cyclin D1[b] | 24 (30%) | 55 (70%) | – |
| All variables[c] (p16/Rb/cyclinD1/p53) | 12 (15%) | 67 (85%) | – |

p53, p53 protein; Rb, retinoblastoma protein; p16, p16$^{INK4a}$ protein. [a]Abnormal = loss of either Rb or p16; [b]Abnormal = loss of Rb or p16, or overexpression of cyclin D1; [c]Abnormal = loss of Rb or p16, or overexpression of cyclin D1; or accumulation of p53 product; [d]Includes a biphenotypic carcinoma with mirror-image areas of Rb and p16 deletion.

immunoreactivity for cyclin D1). This cut-off was selected since normal tissues sometimes showed weak nuclear immunoreactivity for cyclin D1 in less than 5% of nuclei. All negative control sections were verified to be non-reactive.

### Statistical methods

Summary statistics consisted in mean, median, standard deviation for continuous, and number and percentages for categorical variables. Groups were compared by $\chi^2$ test or Fisher's exact test with respect to categorical parameters. The log-rank test was used to compare groups with respect to their survival experience. Since this was an elderly population, only death due to or with disease was treated as endpoint of interest while death due to other causes and survival to the end of the study were considered censoring mechanisms. Survival plots were constructed according to the Kaplan–Meier method. They terminate either with the last death or when there are fewer than ten survivors left. Stepwise Cox regression was applied to determine which combination of parameters would best describe survival, and in particular, to examine whether cyclin D1 provided additional information. All statistical testing was for the two sided alternative. *P*-values less than or equal to 0.05 were considered indicative of a statistically significant effect. No adjustment for multiple testing was made. The statistical software, SAS, on a mainframe computer at the University of Minnesota was used for statistical analyses (SAS Institute Inc., Cary, NC, USA).

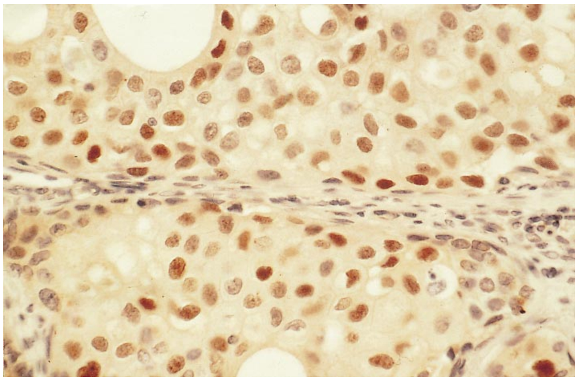

**Figure 3** Example of transitional cell carcinoma with strong overexpression of cyclin D1. In this field, the majority of tumour cells show nuclear reactivity, which ranges in intensity from weak to strong. Original magnification 400×

### RESULTS

Approximately half of the invasive transitional cell carcinomas examined in this series showed loss of either p16$^{INK4a}$ or Rb protein. Twenty-three of 79 carcinomas (29%) showed loss of Rb protein from tumour cell nuclei, while staining for Rb was retained in adjacent normal tissue (endothelial and stromal cells). In three cases, loss of Rb was focal, in that other areas of tumour showed nuclear staining; but stromal cells in the area of negative tumour showed appropriate staining. These cases were counted as Rb-abnormal. Fifteen of 79 carcinomas (all Rb-positive) showed absence of nuclear p16$^{INK4a}$, with p16$^{INK4a}$ immunoreactivity visible in at least some adjacent normal cells (Figure 1A). In general, p16$^{INK4a}$ staining of normal cells and Rb-normal tumours was weak, as previously noted (Geradts et al, 1995) (Figure 1B). By contrast, Rb-deleted carcinomas often showed moderately to markedly increased nuclear staining for p16$^{INK4a}$ (Figure 1C), consistent with previous reports that Rb is a negative regulator of p16$^{INK4a}$ transcription, and that loss of Rb function is associated with elevated levels of p16$^{INK4a}$ protein (Serrano et al, 1993; Li et al, 1994; Parry et al, 1995). One Rb+ carcinoma was equivocal for p16$^{INK4a}$. Finally, one interesting carcinoma showed tumour heterogeneity, with inverse patterns of Rb and p16$^{INK4a}$ loss in separate blocks of tumour (Figure 2). Certain tumour blocks reproducibly showed nuclear Rb immunoreactivity with loss of p16$^{INK4a}$ staining in tumour cells, whereas other blocks reproducibly showed Rb loss in tumour cells with up-regulated p16$^{INK4a}$ expression. The blocks with Rb deletion and retained p16$^{INK4a}$ also showed abnormal accumulation of p53 protein, while Rb+p16$^{INK4a}$-negative blocks did not stain for p53 (Figure 2E,F). Neither area displayed overexpression of cyclin D1.

Overall, 39/79 carcinomas (49%) showed loss of Rb (29%) or p16$^{INK4a}$ (19%), or a mixed pattern of loss of one or the other protein in all blocks tested (1%). One additional carcinoma was p16$^{INK4a}$ equivocal (Rb+). The inverse relationship between loss of p16$^{INK4a}$ and loss of Rb was statistically significant ($P = 0.004$ by Fisher's exact test) (Table 2).

Normal cells either showed no detectable staining for cyclin D1 by immunohistochemistry, or showed weak staining of rare nuclei, with immunoreactivity for cyclin D1 in < 5% of cell nuclei. Among the invasive transitional cell carcinomas comprising our series, 58 (73%) displayed minimal or no staining for cyclin D1,

© 1999 Cancer Research Campaign

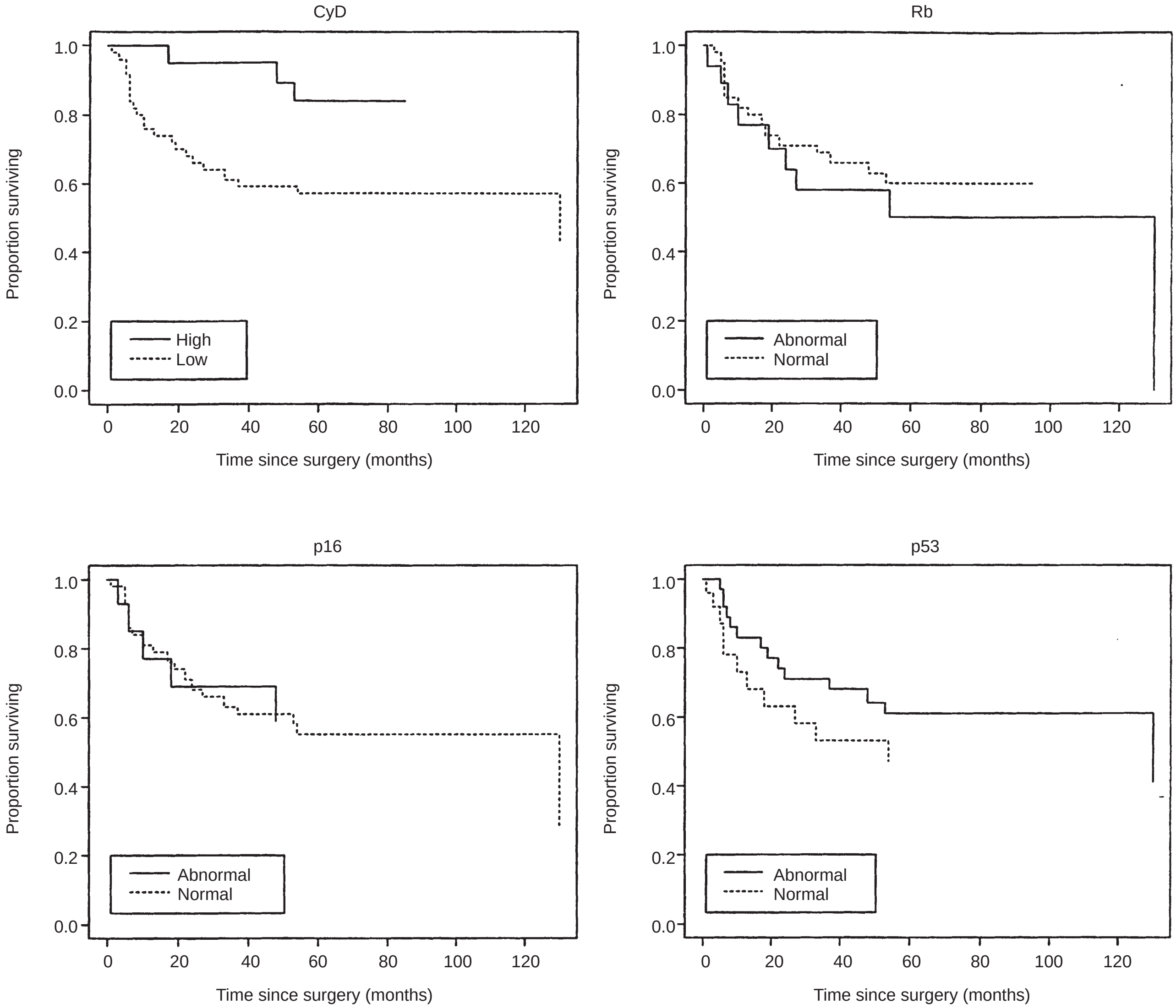

**Figure 4** Kaplan–Meier curves for survival with respect to death of or with disease. Plots terminate when there are fewer than ten patients followed or with the last death. (**A**) Cyclin D1: low (normal) versus high expression; low group contains 58 patients, high group 21 patients. (**B**) Rb: normal (55 patients) versus abnormal (24 patients). (**C**) $p16^{INK4a}$: normal (62 patients) versus abnormal (16 patients). (**D**) p53: normal (29 patients) versus abnormal (50 patients). Cyclin D1 (CyD) $P = 0.02$; for all the other comparisons, $P >$ or = 0.2 according to log-rank statistics while 21 (27%) showed cyclin D1 overexpression with strong staining of 5% or more of tumour cell nuclei (Figure 3). Usually, between 5% and 50% of nuclei were labelled in the cyclin D1 overexpressors. Strong immunoreactivity for cyclin D1 was inversely correlated with loss of Rb protein; all of the 21 cyclin D1 overexpressors were Rb+, while none of the 23 Rb– carcinomas overexpressed cyclin D1. The inverse relationship between cyclin D1 overexpression and Rb protein loss was statistically significant ($P = 0.0002$ by Fisher's exact test) (Table 2). By contrast, cyclin D1 overexpression occurred among $p16^{INK4a}$-negative as well as $p16^{INK4a}$-positive carcinomas. Strong cyclin D1 expression was seen in 6/15 $p16^{INK4a}$-negative carcinomas (40%) as well as in 14/62 $p16^{INK4a}$-conserved tumours (23%) ($P = 0.23$ by $\chi^2$ test). Overall, 55/79 carcinomas (70%) showed abnormalities in Rb, $p16^{INK4a}$, and/or cyclin D1 expression, while 24 carcinomas (30%) had a normal staining pattern for all three proteins (Table 3).

Abnormal p53 accumulation occurred in 50/79 carcinomas (63%). p53 abnormalities were not significantly correlated with Rb, $p16^{INK4a}$, or cyclin D1 status (data not shown). In our series, 12/79 carcinomas (15%) showed no detectable abnormalities in any of the four parameters tested (Table 3).

Using a $\chi^2$ analysis, we tested $p16^{INK4a}$, Rb, cyclin D1 and p53 status for correlation with previously reported variables including TNM stage, grade, DNA ploidy, S phase fraction, percentage of PCNA-positive nuclei, and EGFR status. There was a weak association between Rb loss and an elevated PCNA score ($P = 0.04$). No other associations between PCNA/S phase fraction and Rb/$p16^{INK4a}$/cyclinD1/p53 could be identified in our series; however, only 35 patients had flow histograms interpretable for S phase fraction. High-grade carcinomas (grade 4 out of 4) were more likely to have lost $p16^{INK4a}$ protein than were grade 2 + grade 3 carcinomas (35% v 13% of tumours, $P = 0.027$ by $\chi^2$ test). Grade

© 1999 Cancer Research Campaign

**Table 4** Correlation of histologic grade and expression of p16/Rb or cyclin D1

| | | p16 | | p16 or Rb | | cyclin D1 | |
|---|---|---|---|---|---|---|---|
| | | N | A | N | A | N | A |
| Grade | 2/3 | 49 | 7 | 34 | 22 | 37 | 19 |
| | 4 | 15 | 8 | 6 | 17 | 21 | 2 |
| | | $P = 0.027$ ($\chi^2$) | | $P = 0.006$ ($\chi^2$) | | $P = 0.021$ ($\chi^2$) | |

N = normal expression; A = abnormal expression (loss of expression for Rb; high level of expression for cyclin D1; for p16 or Rb, loss of either protein was counted as abnormal).

**Table 5** Univariate survival analysis

| Potential predictive variable | *P*-value |
|---|---|
| Age (continuous) | 0.9 |
| Grade (2/3 vs 4) | 0.007 |
| TNM Stage[a] (1, 2, 3, 4) | 0.0001 |
| EGFR index[b] (low vs high) | 0.03 |
| Cyclin D1 (low vs high) | 0.02 |
| Rb (normal vs abnormal) | 0.2 |
| p16 (normal vs abnormal) | 0.8 |
| p53 (normal vs abnormal) | 0.3 |
| p16+Rb (normal vs abnormal[c]) | 0.14 |
| p16+Rb+cyclin D1 (normal vs abnormal[d]) | 0.2 |
| All variables (normal vs abnormal[e]) (p16/Rb/cyclinD1/p53) | 0.9 |

EGFR, epidermal growth factor receptor; p53, p53 protein; Rb, retinoblastoma protein; p16, p16$^{INK4a}$ protein. Age (continuous) was analysed by Cox regression. Other variables were analysed by log-rank test. [a]TNM staging was performed according to the American Joint Committee on Cancer (1992), *Manual for staging cancer*, 4th edn; [b]EGFR index was determined in a previous publication (Nguyen et al, 1994); [c]Abnormal = loss of either Rb or p16; [d]Abnormal = loss of Rb or p16, or overexpression of cyclin D1; [e]Abnormal = loss of Rb or p16, or overexpression of cyclin D1, or accumulation of p53 product.

**Table 6** Multivariate survival analysis

| | Coefficient (s.e.m.) | Wald's *P*-value | Relative hazard | Overall significance of model |
|---|---|---|---|---|
| Model 1 | | | | |
| Grade | – | 0.4 | | |
| EGFR | –0.77 (0.42) | 0.07 | 2.16 | |
| CyD | –1.61 (0.74) | 0.03 | 0.2 | $P = 0.003$ |
| Model 2 | | | | |
| TNM stage | 1.283 (0.301) | 0.0001 | 3.61 | |
| CyD | –1.95 (0.75) | 0.009 | 0.14 | $P = 0.0001$ |

Stepwise selection from parameters which had $P < 0.2$ in univariate analyses. Grade: 2 & 3 versus 4. EGFR: epidermal growth factor receptor (low vs high). CyD: cyclin D1 (low vs high). TNM stage (1 vs 2 vs 3 vs 4). TNM stage determined according to the *Manual for Staging of Cancer*, 4th ed. (American Joint Committee on Cancer, 1992).

4 carcinomas were also slightly more likely to show Rb loss than were lower grade carcinomas (39% v 25%, $P = 0.23$). Combining these two findings, high-grade (grade 4) carcinomas were significantly more likely to show deletion of one of these two proteins than were grade 2 + grade 3 carcinomas (74% v 40%, $P = 0.006$ by $\chi^2$ test) (Table 4). Conversely, lower-grade carcinomas were more likely to show strong cyclin D1 overexpression than were grade 4 carcinomas (34% v 9%, $P = 0.021$) (Table 4). Abnormal p53 accumulation was slightly more common among grade 2 + 3 carcinomas, but did not reach statistical significance (68% vs 52%, $P = 0.19$). There were no significant associations between Rb/p16$^{INK4a}$/cyclin D1/p53 status and TNM stage and ploidy. There was a weak association between Rb loss and staining for EGFR ($P = 0.046$). No other significant associations were found between Rb/p16$^{INK4a}$/cyclin D1/p53 and EGFR immunoreactivity.

Disease-specific survival was analysed by log-rank test according to Rb, p16$^{INK4a}$, cyclin D1 and p53 status, the presence or absence of any abnormality in Rb, p16$^{INK4a}$, or cyclin D1, or the presence or absence of any abnormality in any of the four tested variables. Strong cyclin D1 overexpression was associated with better survival ($P = 0.02$ by log-rank test, when compared to weak/negative staining) (Figure 4A). Neither Rb, p16$^{INK4a}$, nor p53 status were significantly correlated with length of survival in this series ($P = 0.2$, 0.8 and 0.3 respectively) (Figure 4 b–d). No statistically significant survival difference was detected between patients whose tumours had a normal phenotype for cyclin D1, Rb, p16$^{INK4a}$ and p53, versus those whose tumours showed one or more abnormalities in these cell cycle proteins (Table 5). The analyses were repeated restricted to muscle-invasive (T2–T4) carcinomas. Again, cyclin D1 overexpression was associated with improved survival ($P = 0.02$, data not shown), while Rb, p16$^{INK4a}$, Rb+p16$^{INK4a}$ and p53 status did not show a significant correlation with outcome ($P = 0.3$, 0.6, 0.6 and 0.3 respectively).

By multivariate analysis, TNM stage and cyclin D1 status were independently associated with disease-specific survival. No other variable significantly affected survival in multivariate analysis (Table 6).

### DISCUSSION

Loss or dysregulation of key components of the G1 cell cycle restriction point is a frequent event in human bladder carcinoma, occurring in two-thirds of the invasive transitional cell carcinomas studied in this series. While loss of Rb protein (Cordon-Cardo et al, 1992; Logothetis et al, 1992; Jahnson et al, 1995; Lipponen and Liukkonen, 1995; Têtu et al, 1996; Cote et al, 1998), deletions of the p16$^{INK4a}$ gene (Orlow et al, 1995; Packenham et al, 1995; Williamson et al, 1995), and cyclin D1 overexpression (Bringuier et al, 1996; Lee et al, 1997; Shin et al, 1997) have previously been reported in transitional cell carcinomas, this is the first large study to examine loss of p16$^{INK4a}$ at the more sensitive protein level, and to document the interrelationship of changes in each of these three components of the G1 checkpoint in the same set of bladder cancers.

Loss of Rb and p16$^{INK4a}$ proteins can occur in malignant cell lines that have retained mRNA expression and do not have obvious abnormalities of the gene at the DNA level. For example, examination of small-cell carcinoma lines revealed structural abnormalities of the Rb gene in only 18% of cell lines, with absence of Rb mRNA in 60% of these cell lines and loss of Rb protein in eight of the nine lines with retained mRNA (Harbour et al, 1988; Horowitz et al, 1990). Similarly, loss of p16$^{INK4a}$ protein was detected in 23/29 tumour cell lines of diverse origin (79%), whereas abnormalities of mRNA expression were identified in only 43% of the same cell lines (Okamoto et al, 1994). Immunohistochemistry offers a means of assessing the presence or loss of Rb and p16$^{INK4a}$ protein within malignant cells using

© 1999 Cancer Research Campaign archival material, with adjacent stroma and normal epithelium serving as internal positive controls for adequacy of antigenic preservation.

We identified loss of Rb protein in 29% of invasive transitional cell carcinomas of the bladder treated by cystectomy (23/79 cases). This correlates well with the frequency of Rb protein loss reported in other series, which has ranged from 16% to 46% (Cordon-Cardo et al, 1992; Logothetis et al, 1992; Jahnson et al, 1995; Lipponen and Liukkonen, 1995; Têtu et al, 1996; Cote et al, 1998). We also identified loss of the $p16^{INK4a}$ tumour suppressor gene product in 15/79 carcinomas (19%). Homozygous deletion of the $p16^{INK4a}$ gene has been reported in 10–22% of primary bladder transitional cell carcinomas (Orlow et al, 1995; Packenham et al, 1995; Williamson et al, 1995). In our series of bladder cancers, loss of Rb and $p16^{INK4a}$ proteins were mutually exclusive events, including one biphenotypic case where several tumour blocks showed a $p16^{INK4a}$+Rb- phenotype, while other tumour blocks showed a mirror image $p16^{INK4a}$-Rb+ phenotype (Figure 1). The $p16^{INK4a}$+Rb− blocks showed strong staining for accumulated p53 protein, while $p16^{INK4a}$−Rb+ blocks were p53-negative, suggesting that this tumour was biclonal. Conventional molecular analysis would have missed this striking example of tumour heterogeneity.

Inverse relationships between loss of $p16^{INK4a}$ and Rb have been previously reported in melanomas (Bartkova et al, 1996), glioblastomas (Ueki et al, 1996), lung carcinomas (Otterson et al, 1994; Shapiro et al, 1995; Kratzke et al, 1996; Sakaguchi et al, 1996), pancreatic carcinomas (Schutte et al, 1997), mesotheliomas (Kratzke et al, 1995), breast carcinomas (Geradts et al, 1995) and a variety of neoplastic cell lines (Aagaard et al, 1995). Double loss of $p16^{INK4a}$ and Rb proteins occurs, but is rare (Otterson et al, 1994; Kratzke et al, 1996). Experimentally, functional Rb protein is required in order for $p16^{INK4a}$ to be able to suppress growth (Lukas et al, 1995*c*; Medema et al, 1995). In Rb-deleted tumours, there may be little selective advantage accruing to cells that also lose $p16^{INK4a}$ (Aagaard et al, 1995).

We identified strong overexpression of cyclin D1 protein in 27% of invasive transitional cell carcinomas of the bladder (21/79 cases). We could not determine by immunohistochemistry alone whether the cyclin D1 gene was amplified or transcriptionally overexpressed. Again, there was an inverse relationship between cyclin D1 overexpression and Rb protein loss. Each of the 21 cyclin D1 overexpressors had retained Rb protein; correspondingly, none of the 23 Rb-negative tumours showed strong cyclin D1 staining. An inverse relationship between cyclin D1 amplification/overexpression and Rb protein loss has also been reported in lung carcinoma cell lines (Schauer et al, 1994) and in oesophageal carcinomas (Jiang et al, 1993). As is the case with $p16^{INK4a}$, experimental studies suggest that loss of Rb protein commits cells to ongoing proliferative cycles regardless of the cellular level of cyclin D1 (Tam et al, 1994; Lukas et al, 1995*b*).

Conversely, we often found cyclin D1 overexpression in $p16^{INK4a}$-negative carcinomas, and vice-versa. Lukas et al have reported that abnormalities of cyclin D1 and $p16^{INK4a}$ often occur concomitantly in human cancer cell lines (Lukas et al, 1995*a*). Experimentally, they were able to demonstrate in human diploid fibroblast strains that microinjection of GST-$p16^{INK4a}$ protein and a neutralizing antibody to cyclin D1 synergistically enhanced G1 arrest, leading them to hypothesize that in Rb-competent cells it is the balance between functional cyclin D1 and $p16^{INK4a}$ which determines whether or not cells will proceed through the G1 checkpoint (Lukas et al, 1995*a*). Our results are consistent with their conclusion that while Rb defects eliminate the G1 checkpoint completely, aberrations of the upstream components such as $p16^{INK4a}$ and cyclin D1 can cooperate in multistep tumorigenesis.

While wild-type p53 has a short half-life and is normally present at low levels in the cell, mutant p53 proteins often form complexes with heat shock protein 70 and accumulate within cells (Finlay et al, 1988). Nuclear immunoreactivity for p53 protein has been used as a surrogate marker for p53 mutation, on the assumption that accumulated p53 protein is likely mutant. Cordon-Cardo et al compared immunohistochemical reactivity for p53 protein in 42 bladder tumours with single-strand conformation polymorphism (SSCP) genetic analysis followed by sequencing, and calculated that immunohistochemistry was 90.3% accurate in detecting p53 mutations (Cordon-Cardo et al, 1994). By immunohistochemistry, we identified abnormal nuclear accumulation of presumably mutant p53 protein in 50/79 invasive bladder carcinomas (63%). There was no association between abnormal p53 staining and abnormalities in the Rb/$p16^{INK4a}$/cyclin D1 pathway.

Univariate survival analysis of our series of 79 invasive transitional cell carcinomas of the bladder treated by cystectomy showed that only TNM stage, grade, EGFR index (from Nguyen et al, 1994) and cyclin D1 overexpression were significantly related to disease-specific survival (Table 5). Unexpectedly, cyclin D1 overexpression was associated with lower grade and better survival, and the favourable prognostic significance of cyclin D1 overexpression persisted in multivariate Cox regression analysis after correction for stage (Table 6). A similar association between cyclin D1 overexpression and favourable outcome has recently been reported for a large series of breast carcinomas (Gillett et al, 1996). Previous studies of bladder carcinoma have suggested that overexpression of cyclin D1 is more common in superficial than invasive bladder neoplasms (Bringuier et al, 1996; Lee et al, 1997). Among superficial bladder tumours, cyclin D1 overexpression has been linked to increased risk for local recurrence but not for tumour invasion (Shin et al, 1997). In our series of invasive carcinomas, the survival advantage of patients with cyclin D1-overexpressing neoplasms did not appear to be due to a lower tumour cell proliferation rate, since cyclin D1 status did not correlate with either S phase fraction or percentage of PCNA-positive nuclei. Instead, it is possible that an increased cellular level of cyclin D1 enhances other processes such as apoptosis which limit tumour growth. In this context, it is interesting that head and neck carcinomas with the highest levels of cyclin D1 expression also display the highest percentage of apoptotic cells when subjected to in-situ end labelling (Kotelnikov et al, 1997). Conversely, bladder carcinomas with G1 checkpoint aberrations other than cyclin D1 overexpression could have a higher frequency of mutations that promote tumour spread (e.g. a pro-metastatic phenotype).

Although high-grade (grade 4) neoplasms were more likely to be deficient in either $p16^{INK4a}$ or Rb protein than were their lower grade counterparts (74% abnormal phenotype for grade 4 carcinomas versus 40% for grade 2/3 carcinomas, $P = 0.006$), neither Rb, $p16^{INK4a}$, nor combined Rb/$p16^{INK4a}$ status reached statistical significance as a predictor of survival in our series. The relationship between $p16^{INK4a}$ protein expression and outcome in bladder carcinoma has not been previously studied, although one report suggested that bladder carcinomas with deletion of the $p16^{INK4a}$ gene are generally of low stage and grade (Orlow et al, 1995). A number of previous papers have reported shorter survival times among patients with Rb-deleted bladder carcinomas (Cordon-Cardo et al, 1992; Logothetis et al, 1992; Lipponen and

 © 1999 Cancer Research Campaign

Liukkonen, 1995; Cote et al, 1998), although others have not found such an association (Jahnson et al, 1995; Têtu et al, 1996). In our series, there was a trend toward poorer outcome for Rb-deleted tumours (Figure 4; *P* = 0.2), which might well have reached significance with a larger cohort. We did not find any link between p16$^{INK4a}$ status and outcome. Based upon previous studies (Lipponen, 1993; Sarkis et al, 1993; Esrig et al, 1994; Cote et al, 1998), we anticipated poorer survival among patients with p53-abnormal tumours, although other groups have published conflicting results (Jahnson et al, 1995; Vet et al, 1995; Glick et al, 1996). We did not find an association between abnormal p53 protein accumulation and poor outcome among our cohort of patients with invasive transitional cell carcinomas treated by cystectomy. We utilized monoclonal antibody D07 to detect p53 accumulation, whereas most previous series, regardless of whether or not they found an association between p53 status and survival, utilized clone p1801. In a previous study (Resnick et al, 1995), we found that D07 and p1801 were equally sensitive in detecting p53 accumulation in paraffin sections from lung and head and neck cancers. It is possible, however, that technical factors played a role in these divergent results. Alternately, differences in patients populations between series (such as relative frequency of early- versus late-stage disease, average patient age, carcinogen exposures, etc.) might have affected both the frequency and prognostic significance of p53 mutation.

One-third of the tumours in our series did not show detectable abnormalities of p16$^{INK4a}$, Rb, or cyclin D1 expression, even though loss of the G1 restriction point appears to be a hallmark of neoplastic disease. These 'normal phenotype' tumours showed no differences in proliferation indices or outcome when compared to tumours that had at least one abnormal staining pattern. One possibility is that some tumours expressed mutated non-functional p16$^{INK4a}$ or Rb that nonetheless was immunoreactive on immunohistochemical analysis. A second possibility is that mutations in other proteins dysregulated the G1 checkpoint complex. For example, mutations in CDK4 have been reported in melanoma cell lines and in familial melanoma kindreds; these mutations prevent the CDK4–cyclin D1 complex from interacting with the inhibitory p16$^{INK4a}$ protein (Bartkova et al, 1996; Zuo et al, 1996; Tsao et al, 1998). Testing for genomic mutations would optimally be performed on fresh or frozen tissue, which is not available for this archival cohort.

In summary, in our series most, although not all, bladder carcinomas had detectable abnormalities in one or more components of the Rb/p16$^{INK4a}$/cyclin D1 cell cycle checkpoint. While cyclin D1 and p16$^{INK4a}$ abnormalities were often seen in the same tumours, we found a strong inverse correlation between p16$^{INK4a}$/cyclin D1 abnormalities and loss of the Rb protein, as has been reported for other neoplasms. Abnormal accumulation of p53 protein was common, occurring in 63% of our cases, and was not correlated with the presence or absence of cyclin D1 overexpression, or p16$^{INK4a}$ and Rb loss. In this series, only TNM stage and cyclin D1 overexpression reached significance as independent predictors of disease-free survival. Cyclin D1 overexpression appeared to occur in a subset of lower grade, less aggressive carcinomas with a relatively favourable prognosis.

### ACKNOWLEDGEMENTS

We appreciate the technical assistance of Ms Pat Rene and the staff of the Minneapolis Department of Veterans Affairs Medical Center Histology Laboratory: Sue Dachel, Josie Robida, Pamela Karbon, Dennis Knapp and Wendy Larson. We also appreciate the immunohistochemical expertise of Clint E Lincoln, who performed the Rb protein immunostains, and of Michael Sakata and Robert Maynard, who performed the p16$^{INK4a}$ immunostains. Supported by a Merit Grant from the Department of Veterans Affairs Medical Research Service (RK), and a grant from the University of North Carolina Research Council (JG).

### REFERENCES

Aagaard L, Lukas J, Bartkova J, Kjerulff A-A, Strauss M and Bartek J (1995) Aberrations of p16INK4 and retinoblastoma tumour-suppressor genes occur in distinct sub-sets of human cancer cell lines. *Int J Cancer* **61**: 115–120

American Joint Committee on Cancer (1992) *Manual for Staging of Cancer*, 4th edn, pp. 195–200. Lippincott: Philadelphia

Bartkova J, Lukas J, Guldberg P, Alsner J, Kirkin AF, Zeuthen J and Bartek J (1996) The p16-cyclin D/Cdk4-pRb pathway as a functional unit frequently altered in melanoma pathogenesis. *Cancer Res* **56**: 5475–5483

Bringuier PP, Tamimi Y, Schuuring E and Schalken J (1996) Expression of cyclin D1 and EMS1 in bladder tumours; relationship with chromosome 11q13 amplification. *Oncogene* **12**: 1747–1753

Cordon-Cardo C, Wartinger D, Petrylak D, Dalbagni G, Fair WR, Fuks Z and Reuter VE (1992) Altered expression of the retinoblastoma gene product: prognostic indicator in bladder cancer. *J Natl Cancer Inst* **84**: 1251–1256

Cordon-Cardo C, Dalbagni G, Saez GT, Oliva MR, Zhang Z-F, Rosai J, Reuter VE and Pellicer A (1994) p53 mutations in human bladder cancer: genotypic versus phenotypic patterns. *Int J Cancer* **56**: 347–353

Cote RJ, Dunn MD, Chatterjee SJ, Stein JP, Shi S-R, Tran Q-C, Hu SX, Xu HJ, Groshen S, Taylor CR, Skinner DG and Benedict WF (1998) Elevated and absent pRb expression is associated with bladder cancer progression and has cooperative effects with p53. *Cancer Res* **58**: 1090–1094

El-Deiry WS, Tokino T, Velculescu VE, Levy DB, Parsons R, Trent JM, Lin D, Mercer WE, Kinzler KW and Vogelstein B (1993) WAF1, a potential mediator of p53 tumor suppression. *Cell* **75**: 817–825

Esrig D, Elmajian D, Groshen S, Freeman JA, Stein JP, Chen S-C, Nichols PW, Skinner DG, Jones PA and Cote RJ (1994) Accumulation of nuclear p53 and tumour progression in bladder cancer. *N Engl J Med* **331**: 1259–1264

Finlay CA, Hinds PW, Tan T-H, Eliyahu D, Oren M and Levine AJ (1988) Activating mutations for transformation by p53 produce a gene product that forms an hsc70-p53 complex with an altered half-life. *Mol Cell Biol* **8**: 531–539

Geradts J, Kratzke RA, Niehans GA and Lincoln CE (1995) Immunohistochemical detection of the cyclin-dependent kinase inhibitor 2/ multiple tumor suppressor gene 1 (CDKN2/MTS1) product p16INK4a in archival human solid tumors: correlation with retinoblastoma protein expression. *Cancer Res* **55**: 6006–6011

Geradts J, Kratzke RA, Crush-Stanton S, Wen SF and Lincoln CE (1996) Wild-type and mutant retinoblastoma protein in paraffin sections. *Mod Pathol* **9**: 339–347

Gillett C, Smith P, Gregory W, Richards M, Millis R, Peters G and Barnes D (1996) Cyclin D1 and prognosis in human breast cancer. *Int J Cancer (Pred Oncol)* **69**: 92–99

Glick SH, Howell LP and White RWD (1996) Relationship of p53 and bcl-2 to prognosis in muscle-invasive transitional cell carcinoma of the bladder. *J Urol* **155**: 1754–1757

Gruis NA, Weaver-Feldhaus J, Liu Q, Frye C, Eeles R, Orlow I, Lacombe L, Ponce-Castaneda V, Lianes P, Latres E, Skolnick M, Cordon-Cardo C and Kamb A (1995) Genetic evidence in melanoma and bladder cancers that p16 and p53 function in separate pathways of tumor suppression. *Am J Pathol* **146**: 1199–1206

Harbour JW, Lai S-L, Whang-Peng J, Gazdar AF, Minna JD and Kaye FJ (1988) Abnormalities in structure and expression of the human retinoblastoma gene in SCLC. *Science* **241**: 353–357

Horowitz JM, Park S-H, Bogenmann E, Cheng J-C, Yandell DW, Kaye FJ, Minna JD, Dryja TP and Weinberg RA (1990) Frequent inactivation of the retinoblastoma anti-oncogene is restricted to a subset of human tumour cells. *Proc Natl Acad Sci USA* **87**: 2775–2779

Jahnson S, Risberg B, Karlsson MG, Westman G, Bergström R and Pedersen J (1995) p53 and Rb immunostaining in locally advanced bladder cancer: relation to prognostic variables and predictive value for the local response to radical radiotherapy. *Eur Urol* **28**: 135–142

Jiang W, Zhang Y-J, Kahn SM, Hollstein MC, Santella RM, Lu S-H, Harris CC, Montesano R and Weinstein IB (1993) Altered expression of the cyclin D1 and retinoblastoma genes in human esophageal cancer. *Proc Natl Acad Sci USA* **90**: 9026–9030

Kotelnikov VM, Coon JS IV, Mundle S, Kelanic S, LaFollette S, Taylor S IV, Hutchinson J, Panje W, Caldarelli DD and Preisler HD (1997) Cyclin D1 expression in squamous cell carcinomas of the head and neck and in oral mucosa in relation to proliferation and apoptosis. *Clin Cancer Res* **3**: 95–101

Kratzke RA, Otterson GA, Lincoln CE, Ewing S, Oie H, Geradts J and Kaye FJ (1995) Immunohistochemical analysis of the p16-INK4a cyclin-dependent kinase inhibitor in malignant mesothelioma. *J Natl Cancer Inst* **87**: 1870–1875

Kratzke RA, Greatens TM, Rubins JB, Maddaus MA, Niewoehner DE, Niehans GA and Geradts J (1996) Rb and p16INK4a expression in resected non-small-cell lung tumours. *Cancer Res* **56**: 3415–3420

Lee CCR, Yamamoto S, Morimura K, Wanibuchi H, Nishisaka N, Ikemoto S, Nakatani T, Wada S, Kishamoto T and Fukushima S (1997) Significance of cyclin D1 overexpression in transitional cell carcinomas of the urinary bladder and its correlation with histopathologic features. *Cancer* **79**: 780–789

Li Y, Nichols MA, Shay JW and Xiong Y (1994) Transcriptional repression of the D-type cyclin-dependent kinase kinase inhibitor p16 by the retinoblastoma susceptibility gene product pRb. *Cancer Res* **54**: 6078–6082

Lipponen PK (1993) Over-expression of p53 nuclear oncoprotein in transitional cell bladder cancer and its prognostic value. *Int J Cancer* **53**: 365–370

Lipponen PK and Liukkonen TJO (1995) Reduced expression of retinoblastoma (Rb) gene protein is related to cell proliferation and prognosis in transitional-cell bladder cancer. *J Cancer Res Clin Oncol* **121**: 44–50

Logothetis CJ, Xu H-J, Ro JY, Hu S-X, Sahin A, Ordonez N and Benedict WF (1992) Altered expression of retinoblastoma protein and known prognostic variables in locally advanced bladder cancer. *J Natl Cancer Inst* **84**: 1256–1261

Lukas J, Aagaard L, Strauss M and Bartek J (1995*a*) Oncogenic aberrations of p16INK4/CDKN2 and cyclin D1 cooperate to deregulate G1 control. *Cancer Res* **55**: 4818–4823

Lukas J, Bartkova J, Rohde M, Strauss M and Bartek J (1995*b*) Cyclin D1 is dispensable for G1 control in retinoblastoma gene-deficient cells independently of cdk4 activity. *Mol Cell Biol* **15**: 2600–2611

Lukas J, Parry D, Aagard L, Mann DJ, Bartkova J, Strauss M, Peters G and Bartek J (1995*c*) Retinoblastoma-protein-dependent cell-cycle inhibition by the tumour suppressor p16. *Nature* **375**: 503–506

Medema RH, Herrera RE, Lam F and Weinberg RA (1995) Growth suppression by p16-ink4 requires functional retinoblastoma protein. *Proc Natl Acad Sci USA* **92**: 6289–6293

Nguyen PL, Swanson PE, Jaszcz W, Aeppli DM, Zhang G, Singleton TP, Ward S, Dykoski D, Harvey J and Niehans GA (1994) Expression of epidermal growth factor receptor in invasive transitional cell carcinoma of the urinary bladder: A multivariate survival analysis. *Am J Clin Pathol* **101**: 166–176

Okamoto A, Demetrick DJ, Spillare EA, Hagiwara K, Hussain SP, Bennett WP, Forrester K, Gerwin B, Greenblatt MS, Serrano M, Shiseki M, Yokota J, Beach DH and Harris CC (1994) p16-INK4 mutations and altered expression in human tumors and cell lines. *Cold Spring Harbor Symp Quant Biol* **59**: 49–57

Orlow I, Lacombe L, Hannon GJ, Serrano M, Pellicer I, Dalbagni G, Reuter VE, Zhang Z-F, Beach D and Cordon-Cardo C (1995) Deletion of the p16 and p15 genes in human bladder tumors. *J Natl Cancer Inst* **87**: 1524–1529

Otterson GA, Kratzke RA, Coxon A, Kim YW and Kaye FJ (1994) Absence of p16INK4 protein is restricted to the subset of lung cancer lines that retains wildtype RB. *Oncogene* **9**: 3375–3378

Packenham JP, Taylor JA, Anna CH, White CM and Devereux T (1995) Homozygous deletions but no sequence mutations in coding regions of p15 or p16 in human primary bladder tumours. *Mol Carcinogen* **14**: 147–151

Parry D, Bates S, Mann DJ and Peters G (1995) Lack of cyclin D-cdk complexes in Rb-negative cells correlates with high levels of p16INK4/MTS1 tumour suppressor gene product. *EMBO J* **14**: 503–511

Resnick JM, Cherwitz D, Knapp D, Uhlman D and Niehans GA (1995) A microwave method that enhances detection of aberrant p53 expression in formalin-fixed, paraffin-embedded tissues. *Arch Pathol Lab Med* **119**: 360–366

Rosai J (1996) *Ackerman's Surgical Pathology*, 8th edn, pp. 1197–1201. Mosby: St Louis

Sakaguchi M, Fujii Y, Hirabayashi H, Yoon H-E, Komoto Y, Oue T, Kusafuka T, Okada A and Matsuda H (1996) Inversely correlated expression of p16 and Rb protein in non-small-cell lung cancers: an immunohistochemical study. *Int J Cancer* **65**: 442–445

Sarkis AS, Dalbagni G, Cordon-Cardo C, Zhang Z-F, Sheinfeld J, Fair WR, Herr HW and Reuter VE (1993) Nuclear overexpression of p53 protein in transitional cell bladder carcinoma: a marker for disease progression. *J Natl Cancer Inst* **85**: 53–59

Schauer IE, Siriwardana S, Langan TA and Sclafani RA (1994) Cyclin D1 overexpression vs. retinoblastoma inactivation: implications for growth control evasion in non-small cell and small cell lung cancer. *Proc Natl Acad Sci USA* **91**: 7827–7831

Schutte M, Hruban RH, Geradts J, Maynard R, Hilgers W, Rabindran SK, Moskaluk CA, Hahn SA, Schwarte-Waldhoff I, Schmiegel W, Baylin SB, Kern SE and Herman JG (1997) Abrogation of the Rb/p16 tumor-suppressive pathway in virtually all pancreatic carcinomas. *Cancer Res* **57**: 3126–3130

Serrano M, Hannon GJ and Beach D (1993) A new regulatory motif in cell-cycle control causing specific inhibition of cyclinD/CDK4. *Nature* **366**: 704–707

Shapiro GI, Edwards CD, Kobzik L, Godleski J, Richards W, Sugarbaker DJ and Rollins BJ (1995) Reciproca Rb inactivation and p16INK4 expression in primary lung cancers and cell lines. *Cancer Res* **55**: 505–509

Shin KY, Kong G, Kim WS, Lee TY, Woo YN and Lee JD (1997) Overexpression of cyclin D1 correlates with early recurrence in superficial bladder cancers. *Br J Cancer* **75**: 1788–1792

Strauss M, Lukas J and Bartek J (1995) Unrestricted cell cycling and cancer. *Nature Med* **1**: 1245–1246

Tam SW, Theodoras AM, Shay JW, Draetta GF and Pagano M (1994) Differential expression and regulation of cyclin D1 protein in normal and tumour human cells: association with Cdk4 is required for cyclin D1 function in G1 progression. *Oncogene* **9**: 2663–2674

Têtu B, Fradet Y, Allard P, Veilleux C, Roberge N and Bernard P (1996) Prevalence and clinical significance of HER-2/neu, p53 and Rb expression in primary superficial bladder cancer. *J Urol* **155**: 1784–1788

Tsao H, Benoit E, Sober AJ, Thiele C and Haluska FG (1998) Novel mutations in the p16/CDKN2A binding region of the cyclin-dependent kinase-4 gene. *Cancer Res* **58**: 109–113

Ueki K, Ono Y, Henson JW, Efird JT, von Deimling A and Louis DN (1996) CDKN2/p16 or RB alterations occur in the majority of glioblastomas and are inversely correlated. *Cancer Res* **56**: 150–153

Vet JAM, Bringuier PP, Schaafsma HE, Witjes JA, Debruyne FMJ and Schalken JA (1995) Comparison of p53 protein overexpression with p53 mutation in bladder cancer: clinical and biologic aspects. *Lab Invest* **73**: 837–843

Williamson MP, Elder PA, Shaw ME, Devlin J and Knowles MA (1995) p16 (CDKN2) is a major deletion target at 9p21 in bladder cancer. *Hum Mol Genet* **4**: 1569–1577

Zuo L, Weger J, Yang Q, Goldstein AM, Tucker MA, Walker GJ, Hayward N and Dracopoli NC (1996) Germline mutations in the p16INK4a binding domain of CDK4 in familial melanoma. *Nat Genet* **12**: 97–99

 *© 1999 Cancer Research Campaign*
